# Evaluation of a multimedia outreach campaign for a multi-target stool DNA test for colorectal cancer screening among non-medicare employer population in the United States

**DOI:** 10.1016/j.pmedr.2022.101848

**Published:** 2022-05-31

**Authors:** Martha E. Shepherd, Ashlee Lecorps, Lori Inman, Lesley-Ann Miller-Wilson

**Affiliations:** a Clinical Medicine and Pediatrics, Vanderbilt University Medical Center, 2694 Fessey Court, Nashville, TN 37204, United States; b Vanderbilt Health at Metro Nashville Public Schools, 2694 Fessey Court, Nashville, TN 37204, United States; c Metro Nashville Public School, United States; d High Fidelity Communications, United States; e Health Economics & Outcomes Research, Exact Sciences Corporation, 441 Charmany Drive, Madison, WI 53719, United States

**Keywords:** Colorectal cancer, Screening, Email campaign, Follow-up colonoscopy, Communication, Outreach

## Abstract

Regular and timely screenings for colorectal cancer (CRC) can improve survival through early cancer detection. The current prospective intervention study assessed the effectiveness of a CRC screening outreach campaign via a multi-media campaign featuring articles in a multi-topic benefits newsletter that was both printed/mailed to homes and emailed to Metro Nashville Public Schools (MNPS) employees and their dependents in the United States. Individuals were included if they were between 45 and 64 years old. The mailed newsletter was sent to 5631 active employees, 868 under 65 retirees, and 4046 retirees with Medicare. The open rate was the highest for the third email (n = 3018; 53.3%). The click-through rate was also the highest for the third email (n = 203;6.7%). Among those who opened at least one of the emails or received a mailed newsletter, 119 members completed the assessment (conversion rate = 3.9%). Among this population, the mt-sDNA completion rate was 64.5% (69 orders completed out of 107 ordered mt-sDNA kits). All 6 patients with a positive mt-sDNA result underwent a follow-up colonoscopy (FU-CY) with the mean (±SD) days to FU-CY among those with positive mt-sDNA test results was 49 (±27) days (median = 42 days). Using emails in conjunction with other targeted interventions to outreach and educate members regarding CRC screening may be an effective strategy to enhance mt-sDNA completion rates.

## 1 Introduction

Routine screenings for colorectal cancer (CRC) can improve survival through early detection of cancer (Siegel et al., 2020). Increasing public awareness through regular reminders, provider recommendations, and patient education may improve CRC screening adherence (Braschi et al., 2014, Beshara et al., 2020, Redmond Knight et al., 2015, Dougherty et al., 2018, Division of Cancer Prevention, 2021). Previous studies have shown a positive impact of multi-media campaigns on CRC screening rates (Dougherty et al., 2018, Jager et al., 2019, Krok-Schoen et al., 2015, Schliemann et al., 2018, Schliemann et al., 2019). However, most published studies focused on interventions such as telephone calls, mailed letters, and automated messages via electronic health records (EHR) systems (Dougherty et al., 2018, Jager et al., 2019, Krok-Schoen et al., 2015). A few studies have examined the impact of email communications sent from healthcare providers (Sequist et al., 2011) and health plans (Muller et al., 2009). These studies showed that emails were as effective as mailed letters or reminders during office visits in improving CRC screening rates. With the growing use and acceptance of using emails for population outreach (Honda and Kagawa-Singer, 2006, Pellino et al., 2017), it is important to understand its effectiveness when combined with other outreach methods. The current study aimed to describe the effectiveness of a CRC screening outreach campaign (email blasts and mailed newsletter) directed at Metro Nashville Public School (MNPS) employees in the United States and assess its impact on mt-sDNA completion rates; as well as evaluate the rates and time to follow-up colonoscopy (FU-CY) among individuals with a positive mt-sDNA test result.

## 2 Methods

### 2.1 Study design

**Fig. 1:**
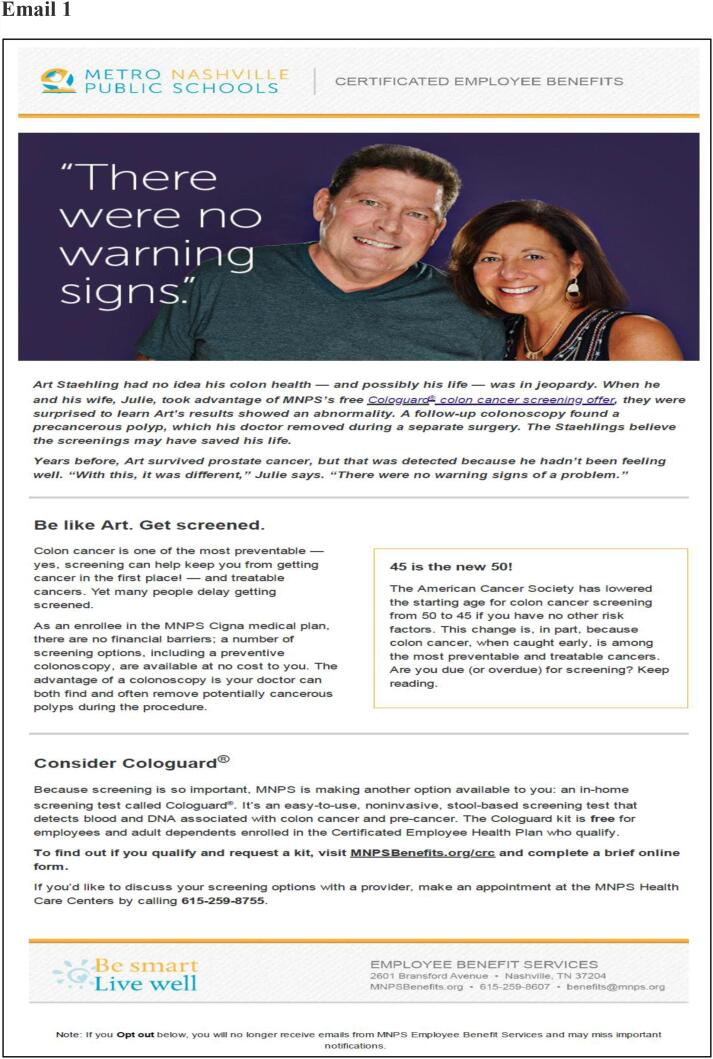

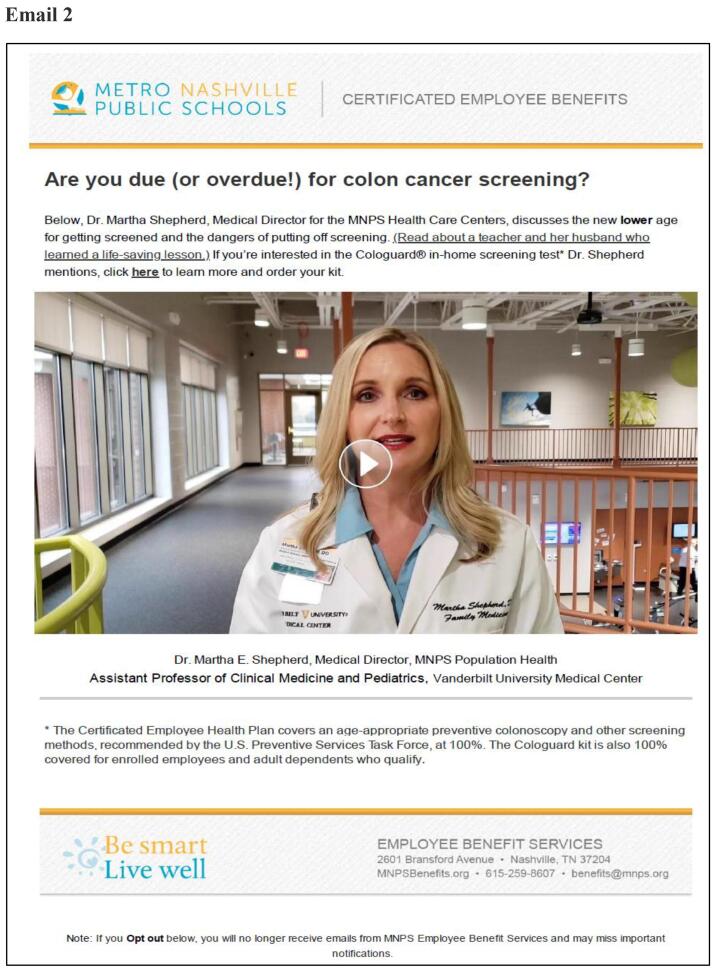

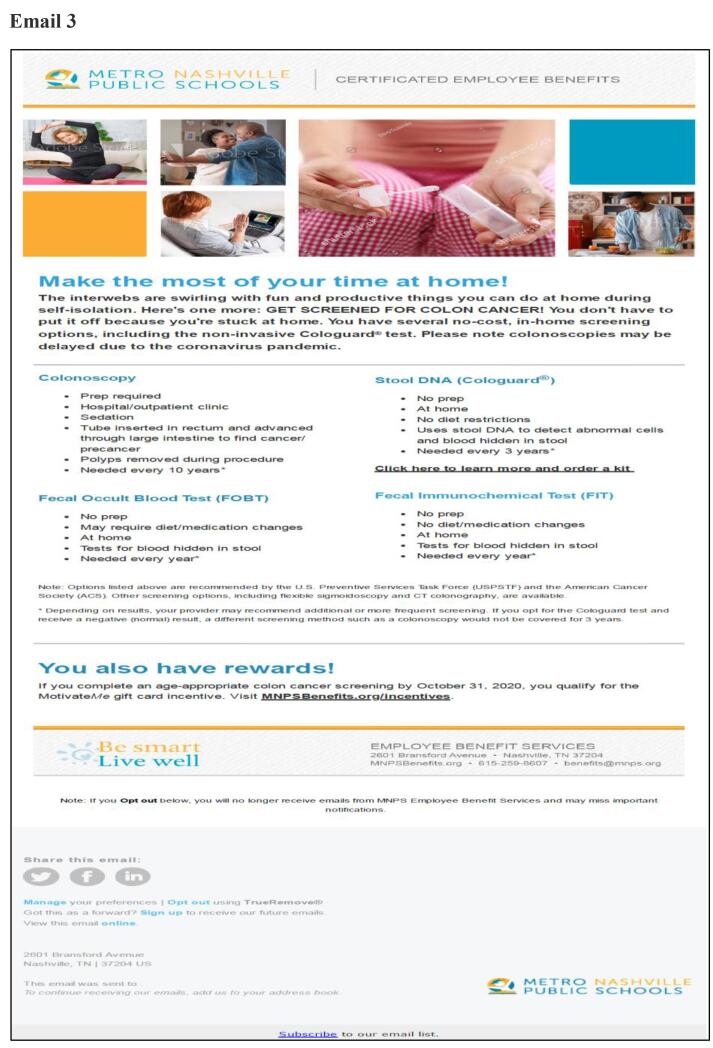
Emails.

This was a prospective study featuring a multi-topic benefits newsletter, which included 3 CRC-focused articles (patient impact story, facts and importance of CRC screening, new age recommendations) (MNPS mailed Newsletter, 2019). The newsletter was both printed/mailed to homes and emailed in the last week of February 2020. The certificated employee email list was updated in between to remove people who quit and add newly eligible individuals. The first email blast was on March 3rd, 2020, and included a patient impact story, provided mt-sDNA indications criteria, and a link to an online assessment form. This was followed by a second email blast on March 12th, 2020, and included CRC facts and the importance of CRC screening, a video featuring the Medical Director from the MNPS employee health clinic, a recap of the mt-sDNA indications criteria, and a link to the online assessment form. Finally, the third email blast was sent on March 25th, 2020, and included information on the impact of early detection making the case for acting now, provided CRC statistics, a recap of mt-sDNA indications criteria, and a link to the online assessment form. The email content can be found in Fig. 1.

Individuals who chose to complete the online assessment form were screened for the study eligibility criteria (described below) by a nurse practitioner from the clinic. The mt-sDNA test kit was ordered and mailed to eligible individuals. Once the stool collection for the test was completed, patients shipped the test back to the Exact Sciences (ES) lab. The test was processed, and the result was provided to the clinic. The staff at the clinic delivered results to the individuals who underwent mt-sDNA testing. If there was a positive mt-sDNA result, the individual was referred for a FU-CY to complete the screening paradigm. The FU-CY after a positive mt-sDNA test was fully covered under the MNPS benefits plan. Data were extracted and compiled from EHRs and other available data sources. Data were de-identified of all protected health information before the analysis. The study was approved by the Vanderbilt Institutional Review Board (IRB#192421).

### 2.2 Study population Inclusion/Exclusion criteria

Individuals were included if they were 45 years and older with average risk to CRC, and were identified as due for CRC screening (defined as no CPT/HCPCS code for: colonoscopy within the last 10 years, gFOBT within the last year, mt-sDNA within the last 3 years, flexible sigmoidoscopy or CT colonography within the last 5 years in their recorded healthcare claims data). Individuals were not considered screening eligible if there was current evidence or history of not being at average risk for CRC, as determined by the presence of at least one International Classification of Diseases (ICD)-9/ICD-10 code indicating the presence, history, or symptoms of any of the following: benign or malignant colorectal neoplasms, colorectal adenomatous polyps, inflammatory bowel disease (ulcerative colitis or Crohn’s disease), family history of CRC or colorectal adenomatous polyps, familial adenomatous polyposis, and hereditary nonpolyposis CRC. Individuals were also required to reside within the state of Tennessee and have a valid phone number so that MNPS providers and the ES lab could contact them. Individuals were excluded if they lost their health plan coverage (or otherwise became ineligible) during the study period or if the initially placed mt-sDNA order was canceled before shipping.

### 2.3 Study measures

Demographic characteristics including age, gender, and race were available for those who ordered the mt-sDNA kit. The study outcomes included email open rate (total emails opened/emails delivered), click-through rate (proportion of email recipients who clicked on the link/emails delivered), email bounce rate (total bounces/emails sent), conversion rate (proportion of email recipients who completed the desired action/emails delivered), and increase in new website visitors during the campaign compared to during the two months prior. The mt-sDNA completion rate was calculated as a proportion of individuals who returned the mt-sDNA kit after it had been shipped to their home and had a test result recorded. Finally, we calculated the proportion of patients who performed FU-CY and time to FU-CY among individuals with a positive mt-sDNA test result from a test ordered between March 4, 2020, and August 31, 2020.

### 2.4 Statistical analysis

Descriptive analyses including means (standard deviations [SDs]), median for continuous variables, and frequency distributions and percentages for categorical variables were performed to describe the study variables. The difference between the email open rates and click-through rates were analyzed with a Bayesian A/B test (Little, 1989).

## 3 Results

The mailed newsletter was sent to 5,631 active employees, 868 under 65 retirees, and 4,046 retirees with Medicare. For the first email blast, a total of 5,708 emails were sent. All the emails were received. The open rate was 40.7% (n = 2,325). The click-through rate was 3.4% (78/2,325). The second email was also sent to the same members. Of those, 2 emails were bounced (0.04%) and 5,706 emails were received. The open rate was 48.3% (n = 2,753) while the click-through rate was 2.8% (n = 161). Between the second and third emails, members who left the plan were removed and new eligible members were added. The third email was sent to 5,660 members/emails. All the emails were received. The open rate was the highest for the third email (n = 3,018; 53.3%; Bayesian probability = 99.8%; posterior expected loss = 0.2%). The click-through rate was also the highest for the third email (n = 203;6.7%; Bayesian probability = 98.7%; posterior expected loss = 3.3%). The number of new website visitors (https://www.mnpsbenefits.org) was increased during the campaign compared to during the 2 months prior.

Among those who opened at least one of the emails or received a mailed newsletter, 119 members completed the assessment (conversion rate = 3.9%). Of those, 12 members did not meet the study inclusion criteria (8 members aged < 45 and 4 members were unable to reach). A total of 107 mt-sDNA kits were ordered by members who received a mailed newsletter and/or an email. The average age of the cohort was 52.4 (±6.3) years. The majority of individuals were females (82.2%), and White (70.1%) (Table 1). Among those who placed the mt-sDNA order, 26 members indicated a mailed newsletter as the mode of reference. Out of 107 ordered mt-sDNA kits, 69 orders were completed (mt-sDNA completion rate = 64.5%). Of those with results, 6 tests were positive. All 6 patients underwent FU-CY. Of these, 4 patients had a finding indicating a polyp removal or a rectal mass excision. The colonoscopy results were unknown for 2 patients. Mean (±SD) days to FU-CY was 49 (±27) days (median = 42 days).

**Table 1:** Demographic Characteristics.

**Measure**	**N**	**%**
**Age (years)**		
Mean (SD); Median	52.4 (6.3); 50.5	
45–49 years	45	42.1%
50–54 years	34	31.8%
55–59 years	15	14.0%
60 years and above	13	12.1%

**Sex**		
Male	19	17.8%
Female	88	82.2%

**Race**		
Black	21	19.6%
White	75	70.1%
Other/Unknown	10	9.3%

## 4 Discussion

This descriptive study evaluated a multi-media outreach program, including a newsletter (sent to 10,545 individuals) and emails (sent to more than 5,500 individuals) to raise mt-sDNA awareness among CRC-average-risk individuals. Note that although the newsletter and emails were sent to the MNPS employees, family members over 45 years were also eligible for screening and could request a kit through the site. We assessed the effectiveness of the campaign using email open rates, click-through rates, and subsequent screening completions. In the healthcare industry, email open rates average 21.7%, and click-through rates average 2.5% (Marketing Benchmarks and Statistics by Industry, 2019). In our study, the email open rates ranged from 40.7% to 53.3%, while the click-through rate was as high as 6.7%.

The third email titled “Make the most of your time at home” had the highest response rate. During this time, the COVID-19 pandemic had just started and most people had to stay home due to work and travel restrictions. This may have caused the higher response rates. In our study, the newsletter was both printed/mailed to homes and emailed in the last week of February 2020. It is safe to assume that all members would have received the newsletter before the second and third email blasts, which may have yielded higher open rates for those as compared to the first email. However, we did not track how many members received/opened the newsletter before vs after the email blasts. Future studies are needed to evaluate if receiving the mailed newsletter prior to the emails helped individuals to be more responsive to the intervention.

During the 6-month-post-intervention period, the mt-sDNA completion rate was 64.5% among those who ordered an mt-sDNA test kit. All individuals with positive test results underwent FU-CY (n = 6) with an average time to colonoscopy being less than 2 months. Prior studies have emphasized the importance of timely colonoscopy after abnormal results on stool-based tests (SBTs) as the risk of CRC-related complications and mortality increases significantly with delays in FU-CY after positive SBTs with the recommended time for FU-CY completion being within nine months of an abnormal SBT (Beshara et al., 2020, San Miguel et al., 2021).

The mt-sDNA completion rate among those who received emails in our study was lower than the CRC screening rates reported among the email intervention group in a previously published study (22.7%) (Muller, et al., 2009). The Muller et al study assessed overall CRC screening while our study focused only on one screening modality. In the Muller et al study, the authors noted that the study population had a higher CRC screening rate than the general HMO population. In another retrospective study among MNPS employees, the mt-sDNA completion rate was 76.8% for office visit-based interactions that included CRC screening reminders by nurse practitioners during office visits (Shepherd et al., 2021). The mt-sDNA completion rate for population-based outreach that included mailed letters followed by mailed mt-sDNA kits to those who agreed to have it shipped to their home was 53.5%. In the current study, the combined outreach with mailed newsletter and email intervention yielded a mt-sDNA completion rate of 64.5% among those who requested a mt-sDNA test, which was higher than the mailed letter intervention but lower than the office-visit based interaction in the retrospective study. These studies suggest the need for a comprehensive approach, combining different interventions to reach and remind patients about their CRC screenings, rather than a single intervention.

There are a few study limitations. First, the study cohort included average-CRC risk non-Medicare MNPS employees and their dependents and the majority were female and white, hence results may not be generalizable to all populations. Participants were predominantly classroom teachers and therefore would have higher education than the average population. Previous studies have shown the inability to leave work for CRC screenings is one of the barriers to screening among the working population (Nagelhout et al., 2017). Further, participants who completed the online assessment were sent the mt-sDNA kits. These individuals may already be highly motivated creating respondent bias. As new members were added to the list before the third email, the increased response rates may be due to increased attention by new members. However, we had access to de-identified data only, hence we were unable to track individual member participation. Participants may have been exposed to CRC screening information from other sources; however, the study team did not have access to those data. Finally, this is a descriptive report and not powered to infer any correlations. Results from this study can be used to design a larger study to compare differences in emails vs. mailed newsletter campaigns.

### CRediT authorship contribution statement

**Martha E. Shepherd:** Conceptualization, Methodology. **Ashlee Lecorps:** Data curation. **Lori Inman:** Data curation. **Lesley-Ann Miller-Wilson:** Supervision, Conceptualization, Methodology, Writing – review & editing.

## Declaration of Competing Interest

The authors declare the following financial interests/personal relationships which may be considered as potential competing interests: Dr. Shepherd, Ms. Lecorps and Ms. Inman have no disclosures to report. Dr. Miller-Wilson is an employee and stockholder of Exact Sciences.
